# Prevalence, Clinical Presentation, and Outcome of Tuberculosis in Patients with Chronic Kidney Disease at a Tertiary Care Hospital in Nepal

**DOI:** 10.1155/2020/7401541

**Published:** 2020-11-01

**Authors:** Ravi R. Pradhan, Mahesh Raj Sigdel

**Affiliations:** ^1^Department of Internal Medicine, Institute of Medicine (IOM), Tribhuvan University Teaching Hospital (TUTH), Kathmandu, Nepal; ^2^Department of Nephrology, Institute of Medicine (IOM), Tribhuvan University Teaching Hospital (TUTH), Kathmandu, Nepal

## Abstract

**Background:**

Tuberculosis (TB) is a serious public health threat in low- and middle-income countries like Nepal. Chronic kidney disease (CKD) patients are at higher risk of developing new infection as well as reactivation of TB. We aimed to determine the prevalence, clinical presentations, and outcome of TB in patients with CKD in Nepal.

**Methods:**

A hospital-based cross-sectional study was performed at Tribhuvan University Teaching Hospital (TUTH), a tertiary level referral centre in Kathmandu, Nepal. We included patients older than 16 years with the diagnosis of CKD stage 3, 4, 5, and 5D (CKD 5 on maintenance dialysis); renal transplant recipients and patients living with HIV/AIDS were excluded. Tuberculosis was diagnosed based on clinical, radiological, and laboratory findings. Prior written informed consent was obtained. Approval was obtained from the Institutional Review Board of the Institute of Medicine. Data entry and statistical analysis were performed using SPSS v21.

**Results:**

A total of 401 patients with CKD were included in the study (mean age, 50.92 ± 17.98 years; 64.8% male). The prevalence of TB in CKD patients was found to be 13.7% (55), out of which 49 were newly diagnosed cases. The most common clinical presentations of TB in CKD were anorexia (85.7%), fever (83.7%), weight loss (51%), and cough (49%). Thirty-eight patients (69.1%) had extrapulmonary TB (EPTB), 12 (21.8%) had pulmonary TB, 3 (5.5%) had disseminated TB, and 2 (3.6%) had miliary TB. Only 4.1% of cases were sputum smear positive. Pleural effusion (34.2%) was the most common EPTB. At 2 months of starting antitubercular therapy, 29 patients out of the 49 newly diagnosed cases of TB (59.2%) had responded to therapy. Mortality at 2 months was 28.6% (14 died amongst 49 patients). Four out of 49 patients (8.2%) did not improve, and 2 (4%) patients were lost to follow-up.

**Conclusion:**

Prevalence and mortality of TB were higher in patients with CKD. Special attention must be given to these people for timely diagnosis and treatment as the presentation is different and diagnosis can be missed.

## 1. Background

Chronic kidney disease (CKD) is a global health problem with estimate that it affects 8–16% of the world's population [1, 2]. It is a major public health problem in Nepal. It is estimated that the prevalence of CKD is around 10.6% in urban areas of Nepal [3].

Tuberculosis (TB) is the second most frequent cause of death from infectious disease worldwide, and its control was one of the millennium development goals [4]. In 2014, a total of 37,025 cases of TB were registered in Nepal. Most of the cases were reported in the middle-aged group, the highest among the 15- to 24-year-old group. Among them, 51% were pulmonary TB and 23% were extrapulmonary. In 2014, the total death from TB was 1049. The overall treatment success rate (all forms) of drug-susceptible TB was 91%, with 1.1% failure rate, 2% defaulted rate, and 3.3% death rate [5]. The disease frequently leads to hospitalization, thus significantly increasing the National Health Service cost.

The incidence of active TB among patients on long-term dialysis is 10 to 25 times higher than that of the general population owing to the immunosuppressant effects of uremia [6]. The rate varies according to regional factors; in developed countries, the incidence ranges between 1.6% and 5.8% [7]. The prevalence of tuberculosis in patients under maintenance dialysis has been reported to be 10.5% [8], 15% [9], and 20% [10] from India, Belgium, and Berlin, respectively. Diagnosing TB in CKD and dialysis patients can be complicated and difficult because of the increased frequency of extrapulmonary involvement, which may result in atypical manifestations and nonspecific symptoms [8, 11]. An increased risk of TB in dialysis patients was first reported by Pradhan et al. [12] in 1974. Impaired immune response in CKD patients may also lead to a delayed response to therapy and increased mortality [8, 11]. Moreover, nutritional status and vitamin D deficiency [13] further contribute to impaired immunity in CKD patients.

Given the globally increasing prevalence of CKD, a merger of CKD and TB epidemics could have significant public health implications in low- to middle-income countries like Nepal. In Nepal, where the burden of TB is high, adequate data on TB in patients with CKD are not available. We set out to study the prevalence, clinical presentations, and outcomes of TB in patients with CKD in a hospital setup.

## 2. Methods

### 2.1. Study Design and Setting

It was a hospital-based, descriptive, observational, cross-sectional study conducted over a period of 12 months (June 2017 to May 2018) at Tribhuvan University Teaching Hospital (TUTH), Institute of Medicine (IOM), Nepal. TUTH is a 700-bedded tertiary care referral hospital located in the capital city, Kathmandu, and provides multispecialty healthcare services to patients from all 77 districts of Nepal. Patients attending the nephrology outpatient department (OPD) and hemodialysis ward and those admitted to the internal medicine and nephrology wards of TUTH were included in the study.

### 2.2. Study Methods

**Figure 1:**
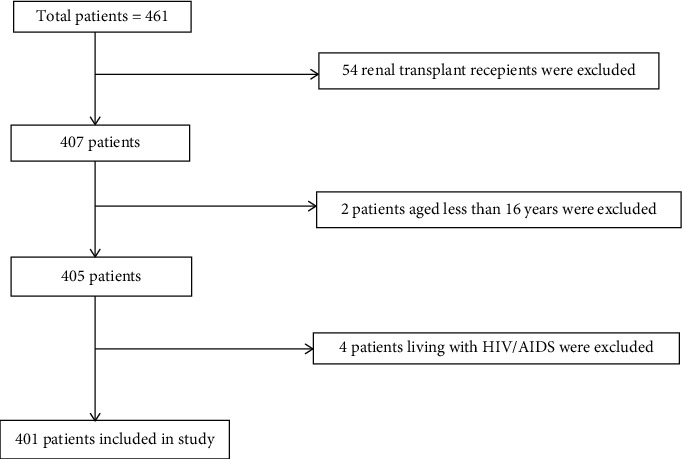
Flowchart showing patients selection based on inclusion and exclusion criteria.

Ethical clearance for the study was obtained from the Institutional Review Board (IRB) of IOM. Written informed consent was taken from all the participants or their legal guardians if patients were under 18 years old. A total of 401 patients meeting the inclusion criteria were included in the study. A diagnosis of CKD was made by the treating physician or nephrologist based on the KDIGO 2012 clinical practice guideline for the evaluation and management of chronic kidney disease (details included in Supplementary material 1) [1]. Cases with confirmed CKD were evaluated, and their stage of CKD was calculated using the CKD-EPI equation [14]. We included patients older than 16 years with the diagnosis of CKD stage 3, 4, 5, and 5D (CKD 5 on maintenance dialysis); renal transplant recipients and patients living with HIV/AIDS were excluded as these could confound our results (details included in Supplementary material 2) (Figure 1).

The diagnosis of tuberculosis was established by the treating physician or nephrologist based on clinical, radiological, microbiological, biochemical, or histological findings (details included in Supplementary material 3) [5, 8, 15]. This study included TB cases which were already diagnosed by the treating physician or nephrologist. Demographic profiles and clinical features were noted, and baseline laboratory and imaging results were recorded in the predesigned proforma. When calculating the prevalence of tuberculosis, the number of previously diagnosed cases and newly diagnosed cases were included. For the clinical features, we collected the current information; we followed up these cases until the duration of admission (if admitted) and until the duration of confirmation of TB diagnosis if they were in OPD and being evaluated for suspected TB. CKD patients with newly diagnosed TB were followed at two months of starting antitubercular therapy (ATT). The patients and their relatives were advised to take/give ATT after dialysis only on the dialysis days. They were assessed at OPD by the treating physician or nephrologist. Those patients who could not attend the OPD were followed up via telephone by the researcher. These patients were assessed for improvement in their symptoms such as subsidence of fever and cough, overall improvement in wellbeing, weight gain, increased appetite, improvement in strength and stamina, or improvement in dyspnea. Those requiring investigations to see for their improvement, for example, regression of lymph node size, pleural effusion, pericardial effusion, ascites, and disappearance of consolidation, went for necessary investigations/assessments as decided by the primary caregiver. Smear-positive TB patients had a repeat acid-fast bacilli (AFB) stain at the completion of two months of ATT.

### 2.3. Definitions of the Variables

The study variables such as current smoker, alcohol consumption, diabetes mellitus, corticosteroid use, immunosuppressive use, and history of contact with TB patients were defined (details included in Supplementary material 4) [16–18].

### 2.4. Sample Size

The sample size was calculated by applying the following formula:

(1) $$\begin{array}{c} n=\frac{{\left(Z_{1-\alpha /2}+Z_{1-\beta /2}\right)}^{2}p\left(1-p\right)}{\varepsilon^{2}}, \end{array}$$

where *n*=number of samples required, *z*=value of the normal deviated considered level of confidence (=1.96), *p*_0_=reference proportion (taken as 0.1) [8], *p*=proportion of interesting outcome (taken as 0.15), alpha (*α*)=a significance level (taken as 0.05), beta (*β*)=a type II error probability (taken as 0.2), and *ε*=*p* − *p*_0_

Using the above formula, the sample size was estimated to be 401.

### 2.5. Statistical Analysis

Continuous variables were expressed as mean ± standard deviation (SD), and categorical variables were expressed as frequency and percentage. The prevalence of TB in patients with CKD was calculated. A *t*-test was employed to compare the mean laboratory values between two groups (CKD patients with newly diagnosed TB and without TB). Bivariate analysis using chi-square test was performed to establish the relationship between TB and its risk factors. Multivariable logistic regression analysis was performed for those variables that were significant in bivariate analysis to independently predict the risk of TB. A *p* value of <0.05 was considered to be statistically significant. Data entry and statistical analysis were performed using SPSS 21 (IBM Corp. Released 2012. IBM SPSS Statistics for Windows, version 21.0. Armonk, NY: IBM Corp.).

## 3. Results

### 3.1. Demographic Characteristics of the Study Population

The mean age of the patients included in the study was 50.92 years (SD = 17.98). There were 260 (64.8%) males; the male-to-female ratio was 1.8 : 1 (Table 1). The demographic profile and etiology of CKD have already been published elsewhere, though chronic glomerulonephritis (36.2%), diabetes mellitus (31.9%), and hypertension (21.7%) were the top three causes of CKD [19].

### 3.2. Prevalence and Clinical Presentation of Tuberculosis in Patients with CKD

The prevalence of tuberculosis in patients with CKD was found to be 13.7% (55 out of 401). Out of 55 cases, 6 were previously diagnosed and were already under ATT, and 49 were newly diagnosed with tuberculosis. The proportion of CKD patients who developed TB was higher in patients receiving MHD for less than one year (41.9%) and session of MHD for one per week (33.3%) (Table 2).

The four most common clinical presentations were decreased appetite (85.7%), fever (83.7%), weight loss (51%), and cough (49%) (Table 3). The duration of clinical presentations in 75.5% (*n* = 37) cases was more than or equal to two weeks and in 24.5% (*n* = 12) cases was less than two weeks.

### 3.3. Types of Tuberculosis in Patients with CKD

**Figure 2:**
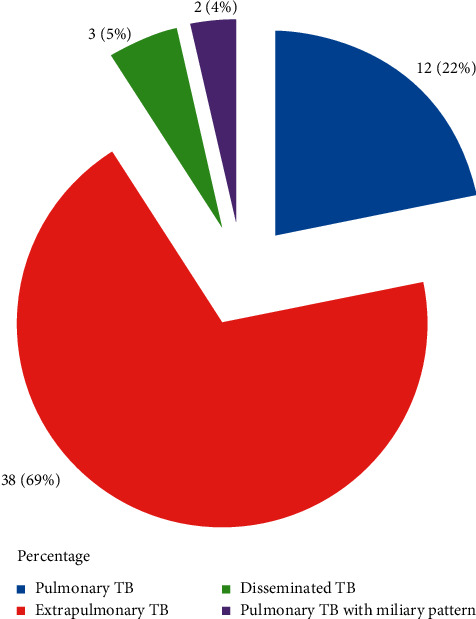
Types of tuberculosis in patients with CKD (*n* = 55).

Extrapulmonary TB (EPTB) (69.1%; *n* = 38) was more common than pulmonary TB (PTB) (21.8%; *n* = 12), followed by disseminated TB (5.5%; *n* = 3) and miliary tuberculosis (3.6%; *n* = 2) (Figure 2).

**Figure 3:**
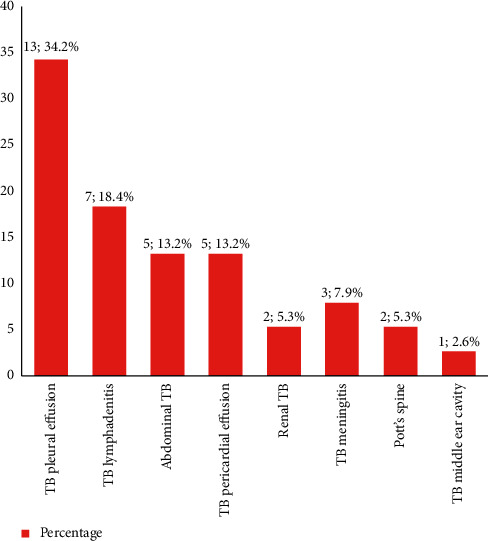
Types of extrapulmonary TB in patients with CKD (*n* = 38).

The most common EPTB was tubercular pleural effusion (34.2%; *n* = 13), followed by TB lymphadenitis (18.4%; *n* = 7), abdominal TB (13.2%, *n* = 5), TB pericardial effusion (13.2%; *n* = 5), renal TB (5.3%, *n* = 2), TB meningitis (7.5%; *n* = 3), Pott's spine (5.3%, *n* = 2), and TB of middle ear cavity (2.6%, *n* = 1) (Figure 3).

The mean duration of CKD diagnosis was 23.46 months (SD = 23) in patients with TB, compared to 15.7 months (SD = 21.8) in CKD patients without TB; this difference was statistically significant (*p* value = 0.02) (Table 1).

### 3.4. Diagnosis of Tuberculosis in Patients with CKD

In the newly diagnosed cases of tuberculosis (*n* = 49), 23 (46.9%) were diagnosed by analysis of body fluid (ascitic, pleural, pericardial, and CSF), 14 (28.6%) by clinical presentations and radiological findings, 6 (12.2%) by FNAC or biopsy, 2 (4.1%) by urine AFB which was further confirmed by urine PCR for *Mycobacterium tuberculosis*, 2 (4.1%) by sputum AFB examination, and 2 (4.1%) by sputum GeneXpert. Taken together, there were only six (12.3%) bacteriologically confirmed cases of tuberculosis.

### 3.5. Comparison of Laboratory Parameters of CKD Patients with and without Tuberculosis

We observed that intact parathyroid hormone (iPTH) was lower and corrected calcium was higher in the newly diagnosed cases of tuberculosis compared to the control group without TB (*p* < 0.05). CKD patients with TB tended to be more anemic and had a lower level of vitamin D and serum albumin compared to CKD patients without TB, though these did not reach statistical significance (*p* > 0.05) (Table 1).

### 3.6. Chest Imaging (Chest Radiograph or CT Scan Chest) and Tuberculin Skin Test (TST) in CKD Patients with Tuberculosis

Out of 49 newly diagnosed cases of TB, 16 (32.7%) had normal chest imaging. The most common abnormal finding was pleural effusion (*n* = 8, 16.3%). Findings of chest imaging are shown in Table 3. TST was positive (>5 mm) only in nine patients (18.4%).

### 3.7. Analysis of Body Fluid (Pleural, Ascitic, Cerebrospinal, and Pericardial)

On analysis of body fluid, it was found that the total leukocyte count was increased with monomorphic predominance. Mean protein and ADA were found to be elevated. Analysis of body fluid is shown in Table 4.

### 3.8. Analysis of Risk Factors Associated with Tuberculosis

On bivariate analysis, we found that corticosteroid, immunosuppressive drugs, and history of contact with TB patients were risk factors for TB compared with their control group without TB (*p* < 0.05). However, we did not find a statistically significant correlation of TB with smoking and alcohol (*p* > 0.05) (Table 5).

On multivariable logistic regression analysis, we found that only history of contact with TB patients (OR: 0.4, 95% CI: 0.2–0.7, *p*=0.004) and corticosteroid use (OR: 5.7, 95% CI: 2.2–14.8, *p*=0.0001) were independent predictors for the development of tuberculosis. Details of the risk factors associated with TB are represented in Table 6.

### 3.9. Outcome of CKD Patients with Tuberculosis at Two Months of Starting ATT

Out of 49 newly diagnosed cases of TB, 29 (59.2%) patients improved at 2 months of starting ATT, 4 (8.2%) did not improve, 14 (28.6%) died during 2 months of starting ATT, and 2 (4%) patients were lost to follow-up.

Seventy percent of the patients (7 out of 10) with pulmonary tuberculosis improved at two months of ATT compared with 62% extrapulmonary TB cases (21 out of 34). None amongst the three cases of disseminated TB improved at two months, while one of the two cases of miliary TB improved and the other one died at two months of starting ATT. The outcomes of TB patients as per different types of TB are presented in Table 7.

The proportion of TB patients who improved at 2 months of ATT was higher in CKD stage 5D (20 out of 31; 64.5%), patients presenting with a duration of symptoms for ≥2 weeks before the diagnosis of TB (24 out of 31; 64.9%), patients receiving MHD for 6 months to one year (6 out of 8; 75%), patients who were not under corticosteroid (26 out of 41; 63.4%) or immunosuppressive drugs (27 out of 46; 58.7%), and patients without history of diabetes mellitus (23 out of 36; 63.9%). Similarly, the mortality in TB patients at 2 months of ATT was higher in CKD stage 4 (4 out of 7; 57.1%), patients presenting with a duration of symptoms for <2 weeks before the diagnosis of TB (4 out of 12; 33.3%), patients receiving MHD for more than one year (2 out of 7; 28.5%), patients under corticosteroid (5 out of 8; 62.5%) or immunosuppressive drugs (1 out of 3; 33.3%), and patients with history of diabetes mellitus (4 out of 13; 30.8%) (Table 8).

## 4. Discussion

CKD is one of the modern-day epidemics of increasing public health importance globally [20]. The prevalence of tuberculosis is still high in developing countries, which are associated with significant morbidity and mortality. Cases of treatment-resistant tuberculosis are increasing [21]. Immunodeficiency is a feature of CKD, making these patients more susceptible to reactivation of TB or new infection.

Patients under MHD living in low- and low-middle-income countries are at increased risk of developing active tuberculosis compared to their counterparts in developed countries. A report from Australia showed that the incidence of TB in CKD patients under MHD was significantly higher in patients born in the highest TB incidence countries (698 per 100,000 per year) compared to those born in low TB incidence countries (18 per 100,000 per year) [22]. This is because active TB results from reactivation of latent tuberculosis infection (LTBI) acquired before dialysis initiation rather than from recent exposure and infection. In our study, we found the prevalence of TB in patients with CKD was 13.7%, which is higher than that reported from India (10.5%) [8], though paradoxically lower to those reported from Belgium (15%) [9] and Germany (20%) [10]. Cough and hemoptysis, the classic symptoms of TB in the general population, are less frequently reported in dialysis patients [8, 11]. In this study, we discovered varied clinical presentation of tuberculosis: decreased appetite (85.7%), fever (83.7%), weight loss (51%), cough (49%), dyspnea (46.9%), chest pain (20.4%), and hemoptysis (4.1%) (Table 3). These features may be attributed to inadequate dialysis, volume overload, uremic symptoms, or complication of hemodialysis in patients with CKD, and this may lead to delay in the diagnosis and treatment of TB, ultimately leading to worse prognosis.

Diagnosis of TB is more complex and difficult in dialysis patients because of atypical clinical presentations, nonspecific symptoms, and increased frequency of extrapulmonary involvement [9, 11]. In our study, extrapulmonary TB was found in 38 patients (69.1%); this observation is consistent with several other studies conducted in India and Turkey [8, 23]. In general population, pulmonary tuberculosis is more common than the extrapulmonary TB; however, in patients with CKD, the ratio is reversed. High prevalence of extrapulmonary TB in patients with CKD is due to impaired cellular immunity with poorly formed granuloma [11]. Different studies have found that the most common form of extrapulmonary TB in patients with CKD is TB lymphadenitis [23]. However, in our study, we found that TB pleural effusion was the predominant type of extrapulmonary TB seen in 34.2% cases amongst all extrapulmonary TB cases, followed by TB lymphadenitis in 18.4%. A similar finding was reported in a prospective study performed in India by Rao et al. [8].

Tuberculosis in patients with CKD remains a diagnostic challenge. In the current study, sputum for AFB was found to be positive only in 4.1% of cases. Tuberculosis was diagnosed in two patients (4.1%) by urine AFB examination and two patients (4.1%) by sputum GeneXpert. Taken together, there were only six (12.3%) bacteriologically confirmed cases of tuberculosis. The main modality of TB diagnosis was based on pathological and biochemical analysis of body fluid (46.9%), which must have required a good amount of clinical judgment without established solid scientific rationale. Since pathological and biochemical analysis of fluid is not available in every centre, it is more challenging to find and treat TB in CKD patients in the peripheries of low-income countries like Nepal. Initial screening with chest imaging seems to be a good investigation modality for the screening of tuberculosis in patients with CKD as 63.7% of CKD patients with TB had abnormal chest imaging in our study. It is highly recommended that the global medical community must put in extra effort and resources to discover newer diagnostic modalities to make a definite diagnosis of tuberculosis.

We discovered that the prevalence of TB was higher in the patients who had received MHD for less than one year (41.9%). Several studies have reported a high frequency of TB cases discovered in the first year of dialysis; this has been attributed to the poor general health at the start of dialysis when host immunity might be most profoundly depressed [23].

On bivariate analysis, we found that corticosteroids, immunosuppressive drugs, and history of contact with TB patients were risk factors for TB. On multivariable logistic regression analysis, we found that only history of contact with TB patients and corticosteroid use was independent predictors of TB. A study done by Chagas et al. discovered that history of contact with TB patients was present in 80% of the patients with latent TB infection [23]. Similarly, corticosteroid as a risk factor for TB was found in the study of Jick et al. [18]. These findings could suggest for isolation of active cases of TB till sputum conversion and judicial use of corticosteroids in advanced stages of glomerulonephritis weighing the risks and benefits of therapy. However, we did not find a statistically significant association of TB with smoking and alcohol consumption. The mean age of TB diagnosis in our study was found to be 44 years, which is comparable to another study conducted by El Kabbaj et al. [24] This young age of TB patients in our study was because the mean age of CKD patients in our study was 50.92 years. However, several other studies showed that hemodialysis patients aged greater than 65 years were at the highest risk of developing TB [25].

CKD patients with TB had significantly higher mean total leukocyte count, corrected calcium, and lower iPTH (*p* < 0.05). Additionally, we discovered that CKD patients with TB had lower hemoglobin, albumin, and vitamin D compared with those without TB; however, these did not reach statistical significance (*p* > 0.05) (Table 1). This observation compares with a recent observational study from the United States which concluded that lower serum albumin is one of the risk factors for TB [26]. A meta-analysis done by Huang et al. [27] showed that vitamin D deficiency is a risk factor for TB. This is because activated vitamin D is required for interferon-*γ*-mediated antimicrobial activity of macrophages against *M. tuberculosis* [27].

In the present study, out of the 49 newly diagnosed cases of tuberculosis, 29 patients (59.2%) improved at 2 months of starting ATT, 4 (8.2%) did not improve, 14 (28.6%) died during 2 months of starting ATT, and 2 (4%) patients lost the follow-up. The mortality rate was relatively higher in miliary TB (one out of two patients died) and disseminated TB (one out of three patients died). The proportion of TB patients who improved at 2 months of ATT was higher in CKD stages 3 (2 out of 3; 66.7%) and 5D (20 out of 31; 64.5%). We postulate that the higher response rate in CKD stage 3 could be because of a lesser degree of immunosuppression and good general health in patients with CKD stage 3 compared to stages 4 and 5. Similarly, the higher response rate in CKD stage 5D could have been attributed to improved general health after starting dialysis. The unusual finding of higher proportion of response rate in patients presenting with a duration of symptoms for ≥2 weeks before the diagnosis of TB and higher mortality in patients presenting with a duration of symptoms for <2 weeks before the diagnosis of TB in our study could have occurred by a chance because of small sample size. Bigger sample size is required before we can draw a definite conclusion regarding this. In literature reviews, no such study has been conducted associating duration of symptoms before diagnosis of TB and mortality.

Studies from the early years of dialysis and some recent reports suggested a high mortality of 17–75% in hemodialysis patients with tuberculosis [11, 12]. The delayed diagnosis and treatment played a major role in some instances. In other cases, the mortality was apparently not caused by the TB itself or its treatment, however, by comorbid conditions. There are reports of favorable outcomes in studies done in Saudi Arabia, most likely due to early diagnosis and treatment [24]. This suggests that if timely diagnosis and intervention are done, patients of CKD with TB can have a better outcome in terms of improved survival and quality of life, which in turn will reduce the hospital stay and total healthcare costs.

To the best of our knowledge, this is the first study of its kind from Nepal. The study has included a reasonable size, nationally representative cohort of CKD patients attending a tertiary care hospital in Nepal. Meticulous documentation of the various sociodemographic profiles, clinical features, investigational findings, and associated medical treatment details followed by rigorous statistical analysis and elaborated discussion of the findings comparing with contemporary literature has been done. The follow-up period of two months for patients initiated on ATT, with only two patients lost to follow-up is good. The high prevalence of tuberculosis in CKD, its risk factors, varied and atypical presentations, diagnostic challenges and limitations compounded by high initial mortality even in treated cases as demonstrated in this study demands for further large-scale discussions and research studies in the field of tuberculosis in general and TB in CKD in particular.

Since the study was cross-sectional, we could not calculate the incidence of tuberculosis in patients with CKD. We evaluated the patients with TB for clinical improvement at two months and assessed their mortality during this period. However, we could not assess their overall response and mortality at the completion of ATT. Since the majority of the diagnosis of TB was clinical, the diagnosis can always be questioned especially when nearly 40% of the patients given ATT either did not respond to therapy or died. The actual cause of death in CKD patients with TB could not be assessed, and from the current study, we cannot say whether this high mortality is due to TB itself or because of other comorbidities. Further, issues related to the clearance of ATT drugs during hemodialysis could not be ascertained. Adherence to therapy could not be stringently verified since the majority of the patients received modified ATT drug dosages due to their renal function and this regimen was not provided by the DOTS scheme of Nepal. Two patients were lost to follow-up, so their clinical improvement at two months of starting ATT could not be assessed.

## 5. Conclusion

The prevalence of tuberculosis in Nepalese patients with CKD was high. Extrapulmonary TB was more common than pulmonary TB; tubercular pleural effusion was the most common form of extrapulmonary tuberculosis. Decreased appetite, fever, weight loss, and cough were the four most common presenting symptoms. At two months of treatment, only 59.2% of cases of tuberculosis responded to ATT. More than a quarter (28.6%) of CKD patients with a diagnosis of tuberculosis died within two months.

We conclude that tuberculosis should be considered in the differential diagnosis of any CKD patient presenting with nonspecific symptoms such as anorexia, fever, and weight loss. An empirical trial with antitubercular medication is justified (especially in endemic areas) in strong clinical suspects if timely definitive diagnosis is not possible. We recommend larger scale, multicenter, multinational studies on tuberculosis in CKD and request the scientific community to develop better tools to confirm the diagnosis of tuberculosis.

## Figures and Tables

**Table 1:** Demographic characteristics, staging, and laboratory parameters of CKD patients with and without tuberculosis.

Parameters	CKD with TB	CKD without TB	*p* value
Age	44 ± 14	52 ± 18	<0.001
Male-to-female ratio	2.7 : 1	1.7 : 1	0.0001
Duration of CKD diagnosis in months	23.46 ± 23	15.7 ± 21.8	0.02

CKD stage			
Stage 3	3	15	0.0001
Stage 4	8	43	0.0001
Stage 5	9	99	0.0001
Stage 5D	35	189	0.0001

Maintenance hemodialysis (MHD)	35	185	0.0001
Peritoneal dialysis	None	4	0.0001

Duration of MHD			
<6 months	17	155	0.0001
6 to 12 months	8	15	0.0001
>12 months	10	15	0.0001

Session of MHD per week			
One/week	1	2	0.25
Two/week	32	175	0.0001
Three/week	2	8	0.0001

Hemoglobin (gm/dL)	8.21	8.6	0.1
Hematocrit (%)	26.46	26.91	0.55
Total leukocyte count (per microlitre)	10267	8679	0.04
Urea (mmol/L)	26.36	27.27	0.61
Creatinine (umol/L)	752.83	781.1	0.64
iPTH (pg/dL)	251.43	328.61	0.036
Phosphorus (mg/dL)	4.76	4.61	0.65
Vitamin D (ng/dL)	24.45	28.48	0.06
Uric acid (umol/L)	425.12	448.92	0.34
Albumin (gm/L)	31.69	33.40	0.06
Corrected calcium (mmol/L)	2.02	1.92	0.02

**Table 2:** Prevalence of TB in different groups.

Parameters	Total number of CKD patients	Number of CKD patients with TB	Within-group prevalence (%)
Duration of dialysis			
Less than 6 months	172	17	9.9
Six months to one year	25	8	32
More than one year	27	10	37

Session of MHD per week			
One	3	1	33.3
Two	207	32	15.5
Three	10	2	20

**Table 3:** Clinical presentation and chest imaging in CKD patients with tuberculosis.

Parameters	Frequency	Percentage
Clinical presentation		
Decreased appetite	42	85.7
Fever	41	83.7
Weight loss	25	51
Cough	24	49
Dyspnea	23	46.9
Chest pain	10	20.4
Hemoptysis	2	4.1
Others	7	14.3

Chest imaging		
Normal	16	32.7
Pleural effusion	8	16.3
Consolidation	1	2.0
Bilateral pulmonary infiltrate	4	8.2
Consolidation with effusion	5	10.2
Unilateral infiltrate	2	4.1
Miliary pattern	2	4.1
Fibrosis in upper lobe with mediastinal lymphadenopathy	4	8.2
Pericardial effusion	5	10.2
Consolidation with tree in bird appearance	1	2.0
Lung abscess	1	2.0

**Table 4:** Analysis of body fluid in patients with TB and CKD.

Parameters (mean)	Pleural fluid (*n* = 12)	Ascitic fluid (*n* = 4)	Cerebrospinal fluid (*n* = 3)	Pericardial fluid (*n* = 4)
Total leukocyte count per *μ*L	1835 ± 1825	583 ± 315	402 ± 647	4910 ± 4784
Polymorph (%)	37 ± 21	27 ± 14	10 ± 17	21 ± 10
Monomorph (%)	63 ± 21	73 ± 14	90 ± 17	71 ± 24
Protein (gm/L)	33.3 ± 6.3	34 ± 7.8	29 ± 4.7	25.2 ± 5.6
LDH (U/L)	522 ± 419	277 ± 67.5	—	—
Sugar (mmol/L)	9 ± 5.8	7.5 ± 3.6	5.1 ± 1	10.4 ± 11.5
ADA (U/L)	32.4 ± 22.8	28 ± 20	11 ± 3.7	31.2 ± 21

**Table 5:** Risk factors associated with tuberculosis.

Parameters	Number of patients with TB (%)	Number of patients without TB (%)	Total	^*∗*^ *p* value
Smoking				
Yes	29 (14.2)	175 (85.8)	204	0.69
No	26 (13.2)	171 (86.8)	197	—

Alcohol				
Yes	28 (13.5)	179 (86.5)	207	0.55
No	27 (14)	168 (86)	194	—

Corticosteroid				
Yes	10 (24.4)	31 (75.6)	41	0.0001
No	45 (12.5)	315 (87.5)	360	—

Immunosuppressive drugs				
Yes	4 (36.4)	7 (63.6)	11	0.0001
No	51 (13)	339 (87)	390	—

History of contact with TB patients				
Yes	16 (25.8)	46 (74.2)	62	0.0001
No	39 (11.5)	300 (88.5)	339	—

Diabetes mellitus				
Yes	13 (10.2)	115 (89.8)	128	0.0001
No	42 (15.4)	231 (84.6)	273	—

^*∗*^ *p* value was calculated using the chi-square test.

**Table 6:** Independent predictors for the development of tuberculosis.

Parameters	OR	95% CI	*p* value
Diabetes mellitus	1.6	0.8–3.2	0.2
History of contact with TB patients	0.4	0.2–0.7	0.004
Corticosteroid	5.7	2.2–14.8	0.0001
Immunosuppressive drugs	0.5	0.09–2.3	0.333

**Table 7:** Outcomes of TB patients at two months of starting ATT as per different types of tuberculosis.

Outcome	Pulmonary TB (%)	Extrapulmonary TB (%)	Disseminated TB (%)	Miliary TB (%)	Total
Improved	7 (70)	21 (62)	0 (0)	1 (50)	29
Not improved	0 (0)	2 (6)	2 (67)	0 (0)	4
Mortality	3 (30)	9 (26)	1 (33)	1 (50)	14
Lost to follow-up	0 (0)	2 (6)	0 (0)	0 (0)	2

Total	10	34	3	2	49

**Table 8:** Factors affecting outcomes of TB patients at two months of starting ATT.

Parameters	Improved (%)	Not improved (%)	Mortality (%)	Lost to follow-up (%)	Total
Duration of symptoms before TB diagnosis					
<2 weeks	5 (41.7)	2 (16.7)	4 (33.3)	1 (8.3)	12
≥2 weeks	24 (64.9)	2 (5.4)	10 (27)	1 (2.7)	37

CKD stage					
Stage 3	2 (66.7)	0 (0)	1 (33.3)	0 (0)	3
Stage 4	3 (42.9)	0 (0)	4 (57.1)	0 (0)	7
Stage 5	4 (50)	0 (0)	3 (37.5)	1 (12.5)	8
Stage 5D	20 (64.5)	4 (13)	6 (19.3)	1 (3.2)	31

Duration of MHD					
Less than 6 months	11 (68.8)	2 (12.5)	2 (12.5)	1 (6.2)	16
6 months to 1 year	6 (75)	0 (0)	2 (25)	0 (0)	8
More than one year	3 (43)	2 (28.5)	2 (28.5)	0 (0)	7

Session of MHD per week					
One	0 (0)	1 (100)	0 (0)	0 (0)	1
Two	19 (65.5)	3 (10.4)	6 (20.7)	1 (3.4)	29
Three	1 (100)	0 (0)	0 (0)	0 (0)	1

History of contact with TB patients					
Yes	10 (66.7)	0 (0)	3 (20)	2 (13.3)	15
No	19 (55.8)	4 (11.8)	11 (32.4)	0 (0)	34

Corticosteroid					
Yes	3 (37.5)	0 (0)	5 (62.5)	0 (0)	8
No	26 (63.4)	4 (9.8)	9 (22)	2 (4.8)	41

Immunosuppressive drugs					
Yes	2 (66.7)	0 (0)	1 (33.3)	0 (0)	3
No	27 (58.7)	4 (8.7)	13 (28.3)	2 (4.3)	46

Diabetes mellitus					
Yes	6 (46.2)	2 (15.4)	4 (30.8)	1 (7.6)	13
No	23 (63.9)	2 (5.6)	10 (27.8)	1 (2.7)	36

## Data Availability

The datasets used and/or analysed during the current study are available from the corresponding author on reasonable request.

## Abbreviations

ACR: Albumin-creatinine ratio
ADA: Adenosine deaminase
AER: Albumin excretion rate
AFB: Acid-fast bacilli
ATT: Antitubercular therapy
CKD: Chronic kidney disease
CKD-EPI: CKD Epidemiology Collaboration
CSF: Cerebrospinal fluid
CT: Computed tomography
DOTS: Directly observed treatment, short-course
EPTB: Extrapulmonary tuberculosis
ESRD: End-stage renal disease
FNAC: Fine-needle aspiration cytology
GFR: Glomerular filtration rate
HIV: Human immunodeficiency virus
IOM: Institute of Medicine
iPTH: Intact parathyroid hormone
IRB: Institutional Review Board
JIOM: Journal of Institute of Medicine
KDIGO: Kidney Disease Improving Global Outcomes
LDH: Lactate dehydrogenase
LTBI: Latent TB infection
MDR-TB: Multidrug-resistant TB
MHD: Maintenance hemodialysis
MRI: Magnetic resonance imaging
OPD: Outpatient department
PCR: Polymerase chain reaction
SD: Standard deviation
TB: Tuberculosis
TST: Tuberculin skin test
TUTH: Tribhuvan University Teaching Hospital
USG: Ultrasonography
ZN: Ziehl Neelsen.

## Supplementary Materials

Supplementary Materials Supplementary material 1: criteria for CKD, markers of kidney damage (one or more), and decreased GFR. Supplementary material 2: inclusion criteria: patients in CKD stage 3 and above with or without maintenance dialysis (hemodialysis or peritoneal dialysis) and age 16 years and above and exclusion criteria: not willing to give consent, age <16 years, CKD with HIV-positive status, and postrenal transplant patients. Supplementary material 3: diagnosis of pulmonary TB, diagnosis of extrapulmonary TB, tubercular pleural effusion, TB lymphadenitis, TB pericardial effusion, TB meningitis, abdominal TB, Pott's spine, diagnosis of disseminated TB, and diagnosis of miliary TB. Supplementary material 4: definitions of the variables .
