# Supplementary material for: Prevalence, Clinical Presentation, and Outcome of Tuberculosis in Patients with Chronic Kidney Disease at a Tertiary Care Hospital in Nepal
Source: Int J Nephrol. 2020 Nov 1;2020:7401541. doi: 10.1155/2020/7401541 (PMC7652626; doi:10.1155/2020/7401541)
Supplement: Supplementary Materials — Supplementary material 1: criteria for CKD, markers of kidney damage (one or more), and decreased GFR. Supplementary material 2: inclusion criteria: patients in CKD stage 3 and above with or without maintenance dialysis (hemodialysis or peritoneal dialysis) and age 16 years and above and exclusion criteria: not willing to give consent, age <16 years, CKD with HIV-positive status, and postrenal transplant patients. Supplementary material 3: diagnosis of pulmonary TB, diagnosis of extrapulmonary TB, tubercular pleural effusion, TB lymphadenitis, TB pericardial effusion, TB meningitis, abdominal TB, Pott's spine, diagnosis of disseminated TB, and diagnosis of miliary TB. Supplementary material 4: definitions of the variables . [file 7401541.f1.docx]

**Supplementary material 1**

**Criteria for CKD**

Either of the following present for ≥ 3 months:^1^

**Markers of kidney damage (one or more)**

- Albuminuria (AER ≥30 mg/24 hours; ACR ≥30 mg/g [ ≥3 mg/mmol])
- Urine sediment abnormalities
- Electrolyte and other abnormalities due to tubular disorders
- Abnormalities detected by histology
- Structural abnormalities detected by imaging
- History of kidney transplantation

**Decreased GFR:** GFR <60 ml/min/1.73 m^2^ (GFR categories G3a–G5)

**Supplementary material 2**

### Inclusion criteria

- Patients in CKD stage 3 and above with or without maintenance dialysis (hemodialysis or peritoneal dialysis)
- Age 16 years and above

### Exclusion criteria

- Not willing to give consent
- Age < 16 years
- CKD with HIV positive status
- Post renal transplant patients

**Supplementary material 3**

**Diagnosis of pulmonary TB:** A diagnosis of pulmonary TB was established by treating physician or nephrologist based on sputum microscopic examination for AFB, sputum for GeneXpert test or on the basis of clinical presentation and chest imaging**.**

**Diagnosis of extra-pulmonary TB:** Diagnosis of extra-pulmonary TB was made as follows:

**Tubercular pleural effusion:** Tubercular etiology was established in presence of clinical features with at least one of the following:^8^

- Pleural fluid protein/serum protein > 0.5
- Pleural fluid lactate dehydrogenase (LDH)/serum LDH > 0.6
- Pleural fluid LDH more than two-thirds of the normal upper limit for serum
- Lymphocyte dominance
- Adenosine deaminase (ADA) > 30 U/L

**TB lymphadenitis:** Diagnosis was established by Fine needle aspiration cytology (FNAC) or excision biopsy (in selected cases when FNAC was inconclusive). Specimen was sent for microscopy, histopathology, and GeneXpert (in selected cases if MDR-TB was suspected).^5^

**TB pericardial effusion:** Clinical features along with some or all of the following findings in the pericardial fluid analysis: increased leukocyte count, lymphocyte dominance, adenosine deaminase >36 U/L. Ziehl Neelsen (ZN) staining for AFB was also done.^8^

**TB meningitis:** Diagnosis was made if cerebrospinal fluid (CSF) analysis revealed increased leukocyte count (> 5 cell/uL), lymphocyte dominance, spider web clot formation on standing, and adenosine deaminase > 6 U/L. Presence of ZN staining for acid- fast bacilli was confirmatory.^8^

**Abdominal TB:** ^5^

TB abdomen was diagnosed if the ascitic fluid analysis showed increased leukocyte count, lymphocyte dominance, low serum ascitic albumin gradient (< 1.1 gm/dl) and high ascitic fluid protein (≥ 2.5 gm/dl), adenosine deaminase > 39 U/L. ZN staining for AFB was also done.

USG abdomen showing intraabdominal fluid (free or loculated), inter-loop ascites, mesenteric lymphadenopathy, bowel wall thickening, enlarged lymph nodes with central necrosis and peripheral enhancement and peritoneal and omental thickening were considered suggestive of tuberculosis.

USG guided FNAC or core biopsy of mesenteric or retroperitoneal lymph node, omentum or peritoneum showing presence of typical findings in histology, microscopy positive for AFB and culture for *M. tuberculosis* would confirm the diagnosis of tuberculosis*.*

**Pott's spine:** Diagnosis was established by MRI spine.^5^

**Diagnosis of disseminated TB:** Disseminated TB was diagnosed if TB involved two or more noncontiguous sites.

**Diagnosis of miliary TB:** Miliary TB was diagnosed by presence of diffuse miliary infiltrate on chest radiograph or high resolution CT scan, or evidence of miliary tubercles in multiple organs at laparoscopy or open surgery.^15^

**Supplementary material 4**

### Definitions of the variables

**Current smoker:** Someone who has smoked greater than 100 cigarettes (including hand rolled cigarettes, cigars, cigarillos etc) in their lifetime and has smoked in the last 28 days.^16^

**Alcohol consumption:** Any consumption of alcohol by the patient irrespective of duration and quantity of alcohol intake.

**Diabetes mellitus:** It was defined as fasting blood sugar fasting plasma sugar ≥ 126 mg/dl or random blood sugar ≥ 200 mg/dl or 2 hour plasma sugar ≥ 200 mg/dl during oral glucose tolerance test or Hemoglobin A_1C_ ≥ 6.5% or on any medications for diabetes mellitus (American Diabetic Association definition) ^17^, or if there was a positive response to the questions “have you ever been told that you have diabetes” or “past history of diabetes.”

**Corticosteroid use:** A current exposure to glucocorticoid was considered if patients had received a prescription for any oral or intravenous glucocorticoid within 120 days prior to the index date. Recent exposure was defined as glucocorticoid use that ended 121–180 days prior to the index date. Use of glucocorticoid 180 days prior to the index date was considered past use.^18^

**Immunosuppressive use:** Patients were considered exposed to an immunosuppressive drug if they have received any of the immunosuppressive drugs (e.g., azathioprine, cyclophosphamide, mycophenolate, tacrolimus, cyclosporine etc) irrespective of dose and duration within one year prior to the diagnosis of TB.

**History of contact with TB patient:** Patients who had shared airspace with a person with infectious TB disease i.e. with family members, friends, coworkers, classmates, and others.
